# Association Between Glycosylated Hemoglobin Level and Cardiovascular Outcomes in Diabetic Patients After Percutaneous Coronary Intervention

**DOI:** 10.1097/MD.0000000000003696

**Published:** 2016-05-13

**Authors:** Jia Zheng, Jing Cheng, Qian Zhang, Cuijuan Qi, Tong Wang, Xinhua Xiao

**Affiliations:** From the Department of Endocrinology (JZ, QZ, CQ, TW, XX), Key Laboratory of Endocrinology, Ministry of Health, Peking Union Medical College Hospital, Diabetes Research Center of Chinese Academy of Medical Sciences & Peking Union Medical College, Beijing; The Key Laboratory of Cardiovascular Remodeling and Function Research (JC), Chinese Ministry of Education and Chinese Ministry of Public Health, Department of Cardiology, Qilu Hospital of Shandong University, Jinan, P.R. China.

## Abstract

Glycosylated hemoglobin (HbA1c) is a critical measure of glycemic control, which may be a reliable predictor of complications after percutaneous coronary intervention (PCI). This systematic review and meta-analysis evaluates the association between HbA1c levels and clinical outcomes in diabetic patients after PCI.

Pubmed, Embase, and Cochrane Library databases (dated to December 2015) were screened for relevant studies. Appropriate diabetic cases and controls, assessed using blood HbA1c levels, were extracted, and statistical analysis was conducted using RevMan 5.3 software. Summary odds ratios (ORs) with 95% confidence intervals (CIs) were used to calculate the associations between HbA1c levels and clinical outcomes in diabetic patients after PCI. Ethics review and approval was not necessary because this systematic meta-analysis did not involve any direct human trials or animal experiments.

Eight studies that reported HbA1c levels for a total of 3290 diabetic subjects after PCI were included in this meta-analysis. Comprehensive integration and analysis revealed a significant correlation between higher HbA1c levels and the risk of target vessel revascularization progression (OR 1.36, 95% CI 1.03–1.82) and nonfatal myocardial infarction after PCI (OR 2.47, 95% CI 1.38–4.44). However, no significant association was found between HbA1c levels and major adverse cardiovascular events (OR 1.02, 95% CI 0.83–1.27), all-cause mortality (OR 0.73, 95% CI 0.52–1.02), cardiac death (OR 1.12, 95% CI 0.62–2.03), or in-stent thrombosis (OR 0.65, 95% CI 0.23–1.87) among diabetic patients after PCI. Sensitivity analysis indicated a statistically robust result and revealed no publication bias.

Our meta-analysis demonstrated that blood HbA1c levels might be associated with higher risks of target vessel revascularization progression and nonfatal myocardial infarction among diabetic patients after PCI. However, further studies with larger sample sizes are required to verify the association.

## Abstract 2

Supplemental Digital Content is available in the text

## INTRODUCTION

Diabetes mellitus (DM) has long been recognized to be an independent risk factor for cardiovascular disease (CVD).^1^ The public health impact of CVD in patients with diabetes has reached epidemic proportions.^1^ Despite recent improvements in medical management and coronary revascularization,^2^ a recent WHO report showed that CVD accounts for approximately 75% of all hospital admissions and approximately 80% of deaths in DM patients. In addition, DM patients undergoing percutaneous coronary intervention (PCI) admitted with acute myocardial infarction (AMI) are more likely to develop stent restenosis, and also major adverse cardiovascular events (MACEs), and have worse clinical outcomes compared with non-DM patients undergoing PCI.^3^

It has been established that glycemic control can impact the clinical outcome in DM patients after PCI. For example, several clinical studies found that admission hyperglycemia (plasma glucose) is associated with increased short and long-term mortality in DM patients with AMI.^4–6^ However, acute stress during AMI at admission can alter glucose metabolism, causing hyperglycemia after an increased catecholamines surge. Thus, these measurements are unstable and unpredictable.^7^ Because of these limitations, chronic abnormal glycometabolic state assessed by blood glycosylated hemoglobin (HbA1c) was utilized to predict the prognosis in DM patients with AMI. HbA1c is a stable marker of long-term blood glucose control, which can reflect the average blood glucose concentrations over the previous 8 to 12 weeks. It is potentially a better prognostic marker of long-term outcome than other glycometabolic parameters reflecting exclusively fasting, postprandial, or incidental glycemia in DM patients.^8,9^

Previous studies have examined the potential association between HbA1c and clinical outcomes among DM patients after PCI.^7,9–16^ However, these surveys were inconclusive and sometimes even contradictory, due to the facts that the majority of studies only enclosed a small sample size resulting in inadequate statistical power to elucidate a positive association or a lack of an association. To overcome this limitation, meta-analysis can be used to synthesize information from varied investigations on the same issue. Hence, we here performed a meta-analysis to evaluate the effects of HbA1c levels on cardiovascular outcomes in DM patients after PCI. To the best of our knowledge, this study is the first meta-analysis to assess the association between HbA1c levels and the progression of clinical outcomes in DM patients after PCI.

## METHODS

This systematic review and meta-analysis was conducted and reported according to Preferred Reporting Items for Systematic Reviews and Meta-analysis (PRISMA) (Supplementary Table 1).

### Data Sources and Searches

Relevant studies were obtained from the Pubmed, Embase, and Cochrane Library databases (dated to December 2015). The main search terms were a combination of Medical Subject Headings terms and text words for DM, HbA1c, cardiovascular outcomes, and PCI. The detailed search terms are shown in the Supplementary Figure 1. All the articles, including conference abstracts, were reviewed. All literature management was performed using Endnote X7 and all references in retrieved articles were scanned to identify other potentially available reports.

### Study Selection Criteria

**FIGURE 1:**
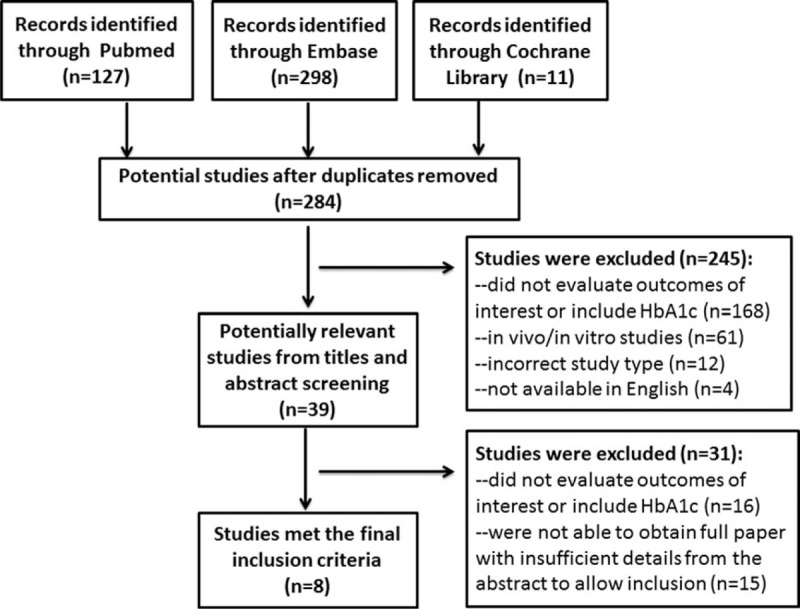
. Study flow chart of trial selection and exclusion.

We included studies that evaluated HbA1c levels and clinical outcomes in diabetic patients after PCI, which were published up to December 2015. The study selection criteria were as follows: studies that measured HbA1C levels; studies that investigated the association between HbA1C levels and cardiovascular outcomes, including MI, target vessel revascularization (TVR), target lesion revascularization (TLR), in-stent restenosis, heart failure, cardiac death, cardiac rehospitalization, stroke, and all-cause mortality; total numbers of the cases and controls could be obtained from articles directly or calculated based on the figures or tables. Study titles and abstracts were initially screened independently by 2 reviewers, and full articles on potentially relevant studies were downloaded and reviewed for inclusion. Disagreement between the 2 reviewers was settled by discussion with a third reviewer. Three reviewers discussed and decided on the final inclusion of studies for this meta-analysis (JZ, JC, and XHX) (Figure 1).

### Data Extraction

Data extraction was performed independently by 2 reviewers (JZ and JC). The following information was extracted from published reports using a standardized protocol and reporting form: last name of first author, year of publication, study design, country of origin, number of enrolled patients, subject characteristics at baseline (age, sex, body mass index, smoking status, hypertension, dyslipidemia and MI, concomitant medication, follow-up duration, and rates) and clinical end-points. Absolute numbers were recalculated when percentages were reported. Disagreement was settled by discussion with a third reviewer (XHX).

### Quality Assessment

Study quality was evaluated systematically by the Newcastle–Ottawa Scale (NOS) (http://www.ohri.ca/programs/clinical_epidemiology/oxford.asp). This system has been developed in which a study is judged on 3 broad perspectives: the selection of the study groups (0–4 points), the comparability of the groups (0–2 points), and the ascertainment of either the exposure or outcome of interest (0–3 points). Disagreement on the score was resolved by discussion with a third reviewer.

### Assessment of Bias Risks

The risks of bias in the included studies were assessed according to the recommended methods of the Cochrane handbook. We evaluated selection bias (including random sequence generation and allocation concealment), performance bias (blinding of participants and personnel), detection bias (blinding of outcome assessment), attrition bias (incomplete outcome data), and reporting bias (selective reporting). Two authors (JZ and JC) independently assessed the risks of bias. Disagreement between the 2 reviewers was settled by discussion with other 2 reviewers (QZ and XHX).

### Data Synthesis and Analysis

Difference was expressed as odds ratios (ORs) with 95% confidence intervals (CIs). The statistical significance of OR was ascertained with Z-test, and *P* < 0.05 was deemed to be statistically significant. The Cochran Q test and *I*^2^ test were all performed to judge the heterogeneity among the studies included in this meta-analysis. Heterogeneity was also considered to be significant at *P* < 0.1 for the Q statistic. *I*^2^ values of 25%, 50%, and 75% corresponded to low, moderate, and high levels of heterogeneity, respectively.^17^ Applying the fixed-effects model or random-effects model depended on the degree of heterogeneity among studies. If there was no evidence of statistical heterogeneity between studies, then a fixed-effects model was used. Otherwise, the random-effects model was adopted.^18^ Sensitivity analysis was carried out by successively excluding the low-quality studies to assess the stability of the outcomes.^19^ Potential publication bias was assessed by visual inspection of the funnel plot, and an asymmetric plot suggested possible publication bias.^20^ Statistical analysis was done using RevMan 5.3 (Nordic Cochrane Center).

## RESULTS

### Studies Included and Participant Characteristics

The selection process of potentially relevant studies is shown in a flow chart (Figure 1). A total of 436 relevant records from electronic databases were identified and 284 were kept after removing duplicates. Thirty-nine potential studies were further reviewed after reading the titles and abstracts. Finally, 8 studies that reported HbA1c levels for a total of 3290 DM subjects after PCI were included in the final meta-analysis.^7,9,11–16^ The characteristics of the included studies are presented in Table 1. These studies were performed in 5 countries (Iran, Japan, Turkey, Poland, and the United States). The mean follow-up duration was about 1 year and the follow-up rates of 7 studies were more than 95%. The enrollment sample size ranged from 142 to 952 subjects. Of the 8 studies, 6 studies indicated that “good-control group” was defined as DM patients with mean HbA1c ≤7%, and “poor-control group” was defined as DM patients with mean HbA1c >7%.^7,9,11,13–15^ The remaining 2 studies showed that the cut-off point of HbA1c was 6.9% and 6.5%, respectively.^12,16^ The 8 studies were mostly with a NOS score of ≥7, which improved the quality of this meta-analysis.^21^

**TABLE 1:**
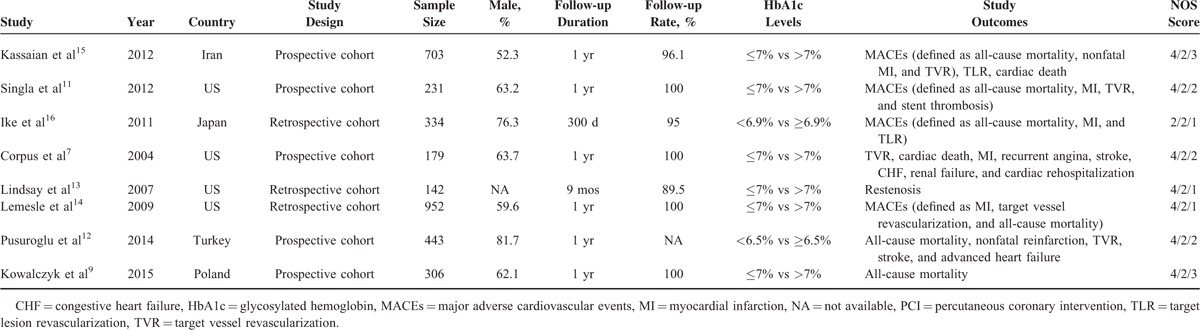
Characteristics of the Studies and Populations Included in this Meta-analysis

### HbA1c Levels and Cardiovascular Outcomes

#### MACEs and All-cause Mortality

**FIGURE 2:**
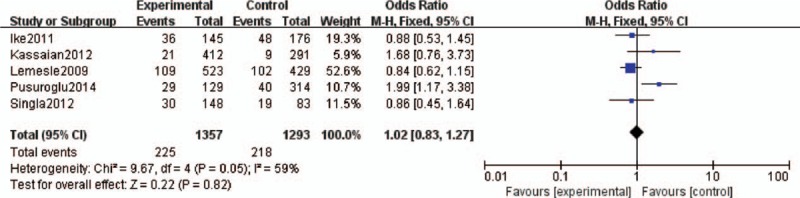
Forest plot of the relationship between HbA1c levels and MACEs. HbA1c = glycosylated hemoglobin, MACEs = major adverse cardiovascular events.

**FIGURE 3:**
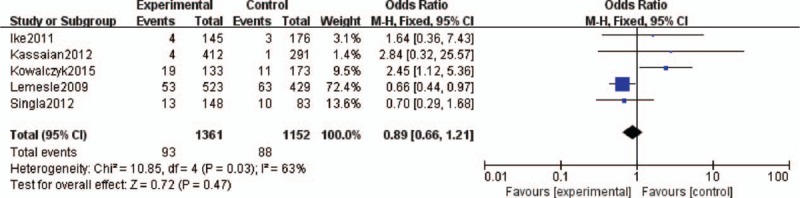
Forest plot of the relationship between HbA1c levels and all-cause mortality. HbA1c = glycosylated hemoglobin.

Five studies assessed the effect of HbA1c levels and MACEs in DM patients after PCI.^11,12,14–16^ No significant association was found between HbA1c levels and MACEs among DM patients after PCI (OR 1.02, 95% CI 0.83–1.27). The heterogeneity was moderate (*I*^2^ = 59%, *P* = 0.05; Figure 2). Five studies assessed the effect of HbA1c levels and all-cause mortality among DM patients after PCI.^9,11,14–16^ No significant association was found between higher HbA1c levels and all-cause mortality (OR 0.73, 95% CI 0.52–1.02). The heterogeneity was very low (*I*^2^ = 0%, *P* = 0.41; Figure 3).

#### TVR and Nonfatal Myocardial Infarction

**FIGURE 4:**
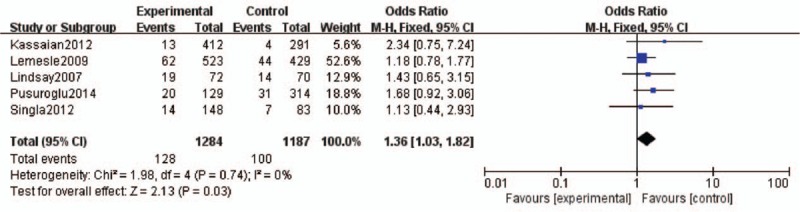
. Forest plot of the relationship between HbA1c levels and TVR. HbA1c = glycosylated hemoglobin, TVR = target vessel revascularization.

**FIGURE 5:**
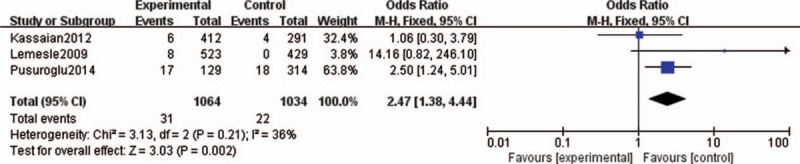
Forest plot of the relationship between HbA1c levels and nonfatal MI. HbA1c = glycosylated hemoglobin, MI = myocardial infarction.

Target vessel revascularization was characterized by ischemia-driven percutaneous or surgical revascularization of the treated vessel. Five studies evaluated the effect of HbA1c levels on the development and progression of TVR among DM patients after PCI.^11–15^ Comprehensive integration and analyses revealed a significant correlation between higher HbA1c levels and the risk of TVR progression (OR 1.36, 95% CI 1.03–1.82). The heterogeneity was low (*I*^2^ = 0%, *P* = 0.74; Figure 4). Three studies assessed the effect of HbA1c levels on the risks of nonfatal MI among DM patients after PCI.^12,14,15^ The analysis revealed that HbA1c levels were significantly associated with the risk of nonfatal MI after PCI (OR 2.47, 95% CI 1.38–4.44). The heterogeneity was low (*I*^2^ = 36%, *P* = 0.21; Figure 5).

#### Cardiac Death and In-stent Thrombosis

Furthermore, considering that cardiac death is a severe complication after PCI, 4 studies were included to assess the effect of HbA1c levels and cardiac death among DM patients after PCI.^7,12,15,16^ There was no significant association between HbA1c levels and the risk of cardiac death (OR 1.12, 95% CI 0.62–2.03). The heterogeneity was very low (*I*^2^ = 0%, *P* = 0.40; Supplementary Figure 2). As part of mechanical injury response, in-stent thrombosis is mainly caused by the effects of vascular smooth muscle cell proliferation and migration, which can occur lately over several months at the location around stent struts by a chronic inflammatory phase.^15^ Two studies were included to assess the effect of HbA1c levels and in-stent thrombosis among DM patients after PCI.^11,16^ No significant association was found between HbA1c levels and stent thrombosis (OR 0.65, 95% CI 0.23–1.87). The heterogeneity was also very low (*I*^2^ = 0%, *P* = 0.43; Supplementary Figure 3).

### Sensitivity Analysis and Publication Bias

To further reinforce our observations, a sensitivity analysis was performed by consecutively excluding individual studies. For HbA1c levels, the corresponding summary ORs were not significantly changed, indicating that our results were statistically robust (detailed data not shown). Funnel plot was designed to visualize a potential publication bias. Funnel plots’ shape of all studies did not reveal obvious evidence of asymmetry, suggesting that no publication bias was observed among studies with pathological indicators.

### Risks of Bias Assessment

**FIGURE 6:**
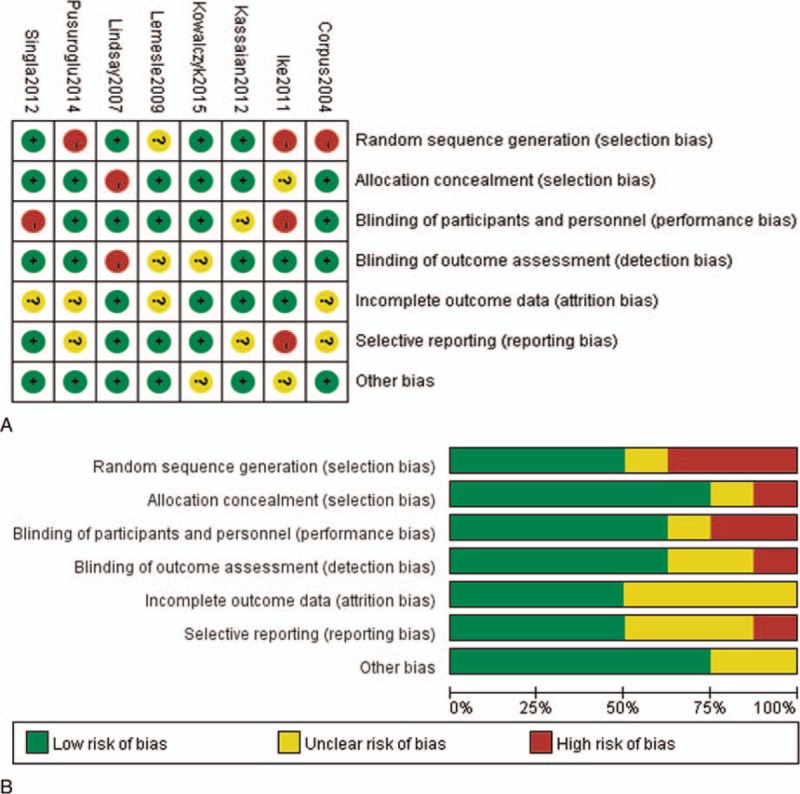
Risks of bias in included studies. A, Risk of bias summary: judgments about each risk of bias item for each included risk. B, Risk of bias graph: judgments about each risk of bias item presented as percentages of all included studies.

The risks of bias assessment of the 8 included studies were summarized in Figure 6. The methods of sequence generation and allocation concealment were used to minimize selection bias in 4 of the studies.^9,11,13,15^ For attrition bias, 4 studies had high follow-up rates and attrition bias was considered to be low risk of bias.^9,13,15,16^ In the other 4 studies, follow-up rate was unclear and judged as unclear bias.^7,11,12,14^ In the included studies, reporting bias was not considered to be a major problem, yet it remains difficult to evaluate it sufficiently.

## DISCUSSION

Diabetes mellitus is a well established risk factor for CVD. To date, HbA1C level assessment is probably the best indicator of long-term glycemia control. Some studies have demonstrated that high HbA1C levels are associated with increased risks of cardiovascular events in DM patients without a history of coronary artery disease.^22,23^ A meta-analysis enrolling 33,040 participants reported that a 0.9% decline in HbA1c level was associated with a 17% decrease in MACEs during acute coronary syndrome in DM patients.^24^ Furthermore, a scientific statement from the American Heart Association and the American Diabetes Association have recommended for the benefit of glycemic control during cardiovascular disease.^25^ However, relatively few studies have investigated the prognostic potential of HbA1c levels in DM patients after PCI, and reported results are conflicting and inconclusive. Therefore, the optimal HbA1C target in DM patients is a subject of ongoing controversy that may be especially important for DM patients with PCI.

In our meta-analysis, 8 studies regarding HbA1c levels and clinical outcomes in DM patients after PCI were selected. Our analysis revealed a significant correlation between higher HbA1c levels and the risk of TVR progression (OR 1.36, 95% CI 1.03–1.82) and nonfatal MI (OR 2.47, 95% CI 1.38–4.44) in DM patients after PCI. However, no significant associations were observed between HbA1c levels and MACEs, all-cause mortality, cardiac death, or in-stent thrombosis. There are several possible mechanisms to explain the associations between higher HbA1c levels and poor clinical outcomes of TVR and nonfatal MI. First, increased HbA1C is a measurement of previous poor glycemic control, and metabolic memory phenomenon^26^ suggests that diabetic cardiovascular diseases can occur even after good glycemic control has been established. Second, there is evidence that chronic hyperglycemia can induce vascular endothelial cell damage, with resultant vasomotor dysfunction, excessive extracellular matrix formation, and increased cellular proliferation,^15^ which can lead to TVR and nonfatal MI after PCI. Third, higher HbA1C levels were associated with some baseline factors, such as dyslipidemia, which can increase the susceptibility to poor cardiovascular outcomes. However, we found no significant associations between HbA1C levels and MACEs. The definition for the MACEs varies among studies, which would be a potential reason for explaining this nonsignificant relationship between HbA1c levels and MACEs. Further studies should be performed to specify the relationship between HbA1c and specific component of MACEs. These data suggest that good glycemic control to obtain lower HbA1c levels may be beneficial in reducing the risks of TVR and nonfatal MI in DM patients undergoing PCI.

However, there are several limitations that should be considered. First, the number of studies and subjects in the studies selected for this meta-analysis were limited. This might limit our statistical power to estimate the associations between HbA1C levels and clinical outcomes. Therefore, a larger size of cohort studies should be needed to acquire a more dependable conclusion. Second, the general follow-up duration is short, ranging from 9 months and 1 year. Thus, a limited follow-up period is 1 potential reason explaining the lack of significant associations between HbA1C levels and all-cause mortality in DM patients. Therefore, studies with longer follow-up duration will be needed. Third, the studies included in this meta-analysis did not consider DM duration, which would be one of the important factors to affect any outcome. DM duration should be taken into consideration in future studies. Fourth, medication history for DM (insulin and oral hypoglycemic agent) and CVD prevention (such as statins, renin–angiotensin system blocking drugs, and beta-blockers) was not accounted in each study, which will influence the clinical outcomes of diabetic patients after PCI. Fifth, publication bias is a major concern in systematic meta-analysis. Most studies are inclined to report positive outcomes, whereas the studies with negative results are often unpublished. In our present study, no evident publication bias was identified. Nevertheless, it is worth noting that the language of published and eligible studies in this meta-analysis was limited to English, which may cause publication bias due to absence of other language studies that met our inclusion criteria. Other limitations inherent to the available literature are the observational nature of studies and the type of the diabetes in some studies.

To the best of our knowledge, our study is the first meta-analysis to assess the prognostic role of HbA1c levels in a population of DM patients after PCI. Despite of the above limitations, this systematic analysis was statistically more convincing than any previous single study. Our meta-analysis strongly suggests that HbA1c levels might be a potential risk factor for TVR and nonfatal MI in DM patients after PCI. In conclusion, our study supports the notion that HbA1c level, a parameter of long-term glycemic control, plays an important role in the prognosis of DM patients undergoing PCI. Meanwhile, to better assess potential associations between HbA1c levels and clinical outcomes among DM patients after PCI, a standardized system, and also interventional studies will be critical to assess the associations between therapies that reduce HbA1c levels and prognosis in DM patients after PCI. Moreover, additional studies should explore the physiopathological mechanisms of HbA1c level and its effects on clinical outcomes.

## Supplementary Material

Supplemental Digital Content

## Supplementary Material

Supplemental Digital Content

## References

[1] Diabetes mellitus: a major risk factor for cardiovascular disease. A joint editorial statement by the American Diabetes Association; The National Heart, Lung, and Blood Institute; The Juvenile Diabetes Foundation International; The National Institute of Diabetes and Digestive and Kidney Diseases; and The American Heart Association. *Circulation* 1999; 100:1132–1133. 10477541 10.1161/01.cir.100.10.1132

[2] Prevention of diabetes mellitus. Report of a WHO Study Group. *World Health Organization technical report series* 1994; 844:1–100. 7941615

[3] Lee TT Feinberg L Baim DS Effect of diabetes mellitus on five-year clinical outcomes after single-vessel coronary stenting (a pooled analysis of coronary stent clinical trials). *Am J Cardiol* 2006; 98:718–721. 16950169 10.1016/j.amjcard.2006.03.059

[4] Ishihara M Kagawa E Inoue I Impact of admission hyperglycemia and diabetes mellitus on short- and long-term mortality after acute myocardial infarction in the coronary intervention era. *Am J Cardiol* 2007; 99:1674–1679. 17560874 10.1016/j.amjcard.2007.01.044

[5] Svensson AM McGuire DK Abrahamsson P Association between hyper- and hypoglycaemia and 2 year all-cause mortality risk in diabetic patients with acute coronary events. *Eur Heart J* 2005; 26:1255–1261. 15821004 10.1093/eurheartj/ehi230

[6] Hadjadj S Coisne D Mauco G Prognostic value of admission plasma glucose and HbA in acute myocardial infarction. *Diabetic Med* 2004; 21:305–310. 15049930 10.1111/j.1464-5491.2004.01112.x

[7] Corpus RA George PB House JA Optimal glycemic control is associated with a lower rate of target vessel revascularization in treated type II diabetic patients undergoing elective percutaneous coronary intervention. *J Am Coll Cardiol* 2004; 43:8–14. 14715174 10.1016/j.jacc.2003.06.019

[8] Diagnosis and classification of diabetes mellitus. *Diabetes Care* 2012; (35 Suppl 1):S64–S71. 22187472 10.2337/dc12-s064 PMC3632174

[9] Kowalczyk J Mazurek M Zielinska T Prognostic significance of HbA1c in patients with AMI treated invasively and newly detected glucose abnormalities. *Eur J Prevent Cardiol* 2015; 22:798–806. 10.1177/2047487314527850 24618476

[10] Tandjung K van Houwelingen KG Jansen H Comparison of frequency of periprocedural myocardial infarction in patients with and without diabetes mellitus to those with previously unknown but elevated glycated hemoglobin levels (from the TWENTE Trial). *Am J Cardiol* 2012; 110:1561–1567. 22939581 10.1016/j.amjcard.2012.07.019

[11] Singla A Orshaw P Boura J Glycosylated hemoglobin and outcomes in diabetic patients with acute myocardial infarction after successful revascularization with stent placement: findings from the guthrie health off-label stent (GHOST) investigators. *J Interven Cardiol* 2012; 25:262–269. 10.1111/j.1540-8183.2011.00715.x 22376172

[12] Pusuroglu H Akgul O Cakmak HA Long-term prognostic value of admission haemoglobin A1c (HbA1c) levels in patients with ST-segment elevation myocardial infarction undergoing primary percutaneous coronary intervention. *Adv Interven Cardiol* 2014; 10:166–174. 10.5114/pwki.2014.45143 PMC4252307 25489302

[13] Lindsay J Sharma AK Canos D Preprocedure hyperglycemia is more strongly associated with restenosis in diabetic patients after percutaneous coronary intervention than is hemoglobin A1C. *Cardiovasc Revascul Med* 2007; 8:15–20. 10.1016/j.carrev.2006.10.002 17293264

[14] Lemesle G Bonello L de Labriolle A Prognostic value of hemoglobin A1C levels in patients with diabetes mellitus undergoing percutaneous coronary intervention with stent implantation. *Am J Cardiol* 2009; 104:41–45. 19576319 10.1016/j.amjcard.2009.02.060

[15] Kassaian SE Goodarzynejad H Boroumand MA Glycosylated hemoglobin (HbA1c) levels and clinical outcomes in diabetic patients following coronary artery stenting. *Cardiovasc Diabetol* 2012; 11:82 1–10. 22805289 10.1186/1475-2840-11-82 PMC3444922

[16] Ike A Nishikawa H Shirai K Impact of glycemic control on the clinical outcome in diabetic patients with percutaneous coronary intervention: from the FU-registry. *Circ J* 2011; 75:791–799. 21427500 10.1253/circj.cj-10-0474

[17] Higgins JP Thompson SG Deeks JJ Measuring inconsistency in meta-analyses. *BMJ (Clin Res ed)* 2003; 327:557–560. 10.1136/bmj.327.7414.557 PMC192859 12958120

[18] Dickersin K Berlin JA Meta-analysis: state-of-the-science. *Epidemiol Rev* 1992; 14:154–176. 1289110 10.1093/oxfordjournals.epirev.a036084

[19] Chootrakool H Shi JQ Yue R Meta-analysis and sensitivity analysis for multi-arm trials with selection bias. *Stat Med* 2011; 30:1183–1198. 21538449 10.1002/sim.4143

[20] Sterne JA Egger M Smith GD Systematic reviews in health care: Investigating and dealing with publication and other biases in meta-analysis. *BMJ (Clin Res ed)* 2001; 323:101–105. 10.1136/bmj.323.7304.101 PMC1120714 11451790

[21] Cheng D Fei Y Liu Y HbA1C variability and the risk of renal status progression in diabetes mellitus: a meta-analysis. *PloS One* 2014; 9:e115509. 25521346 10.1371/journal.pone.0115509 PMC4270779

[22] Wang P Huang R Lu S HbA1c below 7% as the goal of glucose control fails to maximize the cardiovascular benefits: a meta-analysis. *Cardiovasc Diabetol* 2015; 14:124 1–15. 26392171 10.1186/s12933-015-0285-1 PMC4578327

[23] Selvin E Marinopoulos S Berkenblit G Meta-analysis: glycosylated hemoglobin and cardiovascular disease in diabetes mellitus. *Ann Intern Med* 2004; 141:421–431. 15381515 10.7326/0003-4819-141-6-200409210-00007

[24] Ray KK Seshasai SR Wijesuriya S Effect of intensive control of glucose on cardiovascular outcomes and death in patients with diabetes mellitus: a meta-analysis of randomised controlled trials. *Lancet* 2009; 373:1765–1772. 19465231 10.1016/S0140-6736(09)60697-8

[25] Buse JB Ginsberg HN Bakris GL Primary prevention of cardiovascular diseases in people with diabetes mellitus: a scientific statement from the American Heart Association and the American Diabetes Association. *Circulation* 2007; 115:114–126. 17192512 10.1161/CIRCULATIONAHA.106.179294

[26] Nathan DM Cleary PA Backlund JY Intensive diabetes treatment and cardiovascular disease in patients with type 1 diabetes. *N Engl J Med* 2005; 353:2643–2653. 16371630 10.1056/NEJMoa052187 PMC2637991

