# Supplementary material for: Association Between Glycosylated Hemoglobin Level and Cardiovascular Outcomes in Diabetic Patients After Percutaneous Coronary Intervention
Source: Medicine (Baltimore). 2016 May 13;95(19):e3696. doi: 10.1097/MD.0000000000003696 (PMC4902553; doi:10.1097/MD.0000000000003696)

Supplementary Figure 1-search term

**Supplementary Data**

**Supplementary Figure 1**

Databases: Pubmed, Embase and Cochrane Library (dated to December 2015)

Search Terms:

1. diabetes mellitus.ab,ti (The restrictions are ab.ti which refer to the presence of the requested search term in the abstract or title).

2. (Coronary Intervention, Percutaneous OR Coronary Interventions, Percutaneous OR Intervention, Percutaneous Coronary OR Interventions, Percutaneous Coronary OR Percutaneous Coronary Interventions OR Percutaneous Coronary Revascularization OR Coronary Revascularization, Percutaneous OR Coronary Revascularizations, Percutaneous OR Percutaneous Coronary Revascularizations OR Revascularization, Percutaneous Coronary OR Revascularizations, Percutaneous Coronary) .ab,ti.

3. (Myocardial infarction OR Heart attack OR Ischemic heart disease OR Ischaemic heart disease OR Acute coronary syndrome OR Coronary artery disease OR Target vessel revascularization OR Target lesion revascularization OR Major adverse cardiovascular event OR Stent thrombosis OR Restenosis OR Heart failure OR heart failure OR Cardiac failure OR Death OR Mortality ).ab,ti.

4. (A1c OR HbA1c OR glycosylated hemoglobin A1c OR glycosylated haemoglobin A1c OR glycaemic control OR glycemic control).ab,ti.

5. 1 AND 2 AND 3 AND 4

6. Remove duplicates from 5

Supplementary Figure 2: Forest plot of the association between HbA1c levels and cardiac death.


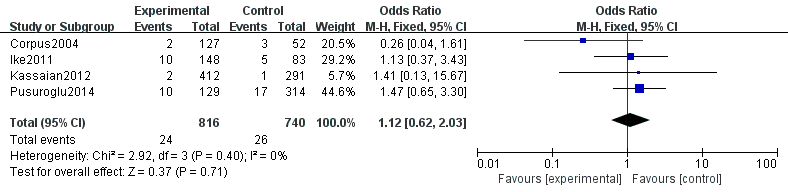


Supplementary Figure 3: Forest plot of the association between HbA1c levels and in-stent thromosis.


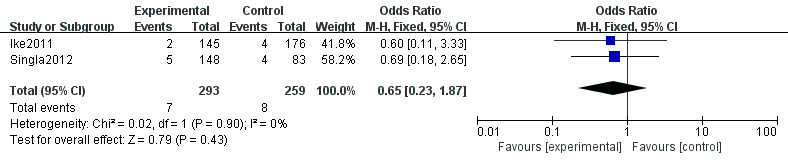

Supplement: Supplemental Digital Content [file medi-95-e3696-s002.doc]
